# New Insights into Unusual Genetic Disorder Pave the Way for Promising Treatments

**DOI:** 10.1371/journal.pbio.1001628

**Published:** 2013-08-13

**Authors:** Janelle Weaver

**Affiliations:** Freelance Science Writer, Carbondale, Colorado, United States of America

Tuberous sclerosis complex (TSC) may not receive as much attention as Down syndrome or other genetic diseases that affect brain development. But as its lengthy name suggests, it is a multifaceted disorder that can have wide-ranging effects on an individual's life. TSC affects about 1 in 6,000 people, causing seizures, mental retardation, and benign tumors in the brain and other organs. Moreover, about one-third of children with TSC meet criteria for autism spectrum disorder. TSC is caused by mutations in two genes known as TSC1 and TSC2, leading to increased activity in the mammalian target of rapamycin (mTOR) signaling pathway, which regulates neuronal signaling and other important cellular processes.

**Figure:**
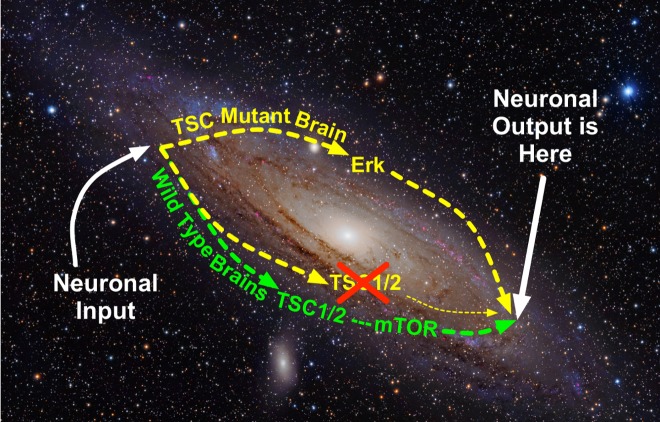
Whereas normal adult mice use the mTOR pathway for the generation of Long Term Depression (LTD) after stimulation of metabotropic Glutamate Receptor 5 (mGluR5), TSC2 mutant mice sidestep the tuberous sclerosis mutation by invoking the Erk signaling pathway. **Thus the LTD in the mouse model of tuberous sclerosis looks outwardly normal but is biochemically distinct.** Image credit: Adapted by Avtar Roopra from the original image by Adam Evans https://en.wikipedia.org/wiki/File:Andromeda_Galaxy_(with_h-alpha).jpg.

Although mTOR inhibitors and other drugs can control symptoms, such as tumors and seizures, these medications sometimes cause serious side effects. A better understanding of the underlying causes of the disorder could lead to the development of more effective treatments. As reported in this issue of *PLOS Biology*, Avtar Roopra of the University of Wisconsin-Madison and his team have made tremendous strides toward this goal by revealing novel signaling pathways involved in TSC.

The researchers found that adult mice with a TSC2 mutation make use of unusual signaling pathways for long-term depression (LTD)—a cellular process that normally relies on the mTOR pathway and is thought to be important for learning and memory. Drug treatments that inhibited these overactive pathways fixed the molecular abnormality, reduced seizure-like activity in brain slices, and corrected an autistic-like behavioral problem in the mutant mice. These findings open up promising new avenues for the treatment of a wide range of TSC symptoms.

Previous studies had shown that juvenile mice with a TSC2 mutation exhibit reduced LTD in the hippocampus—a brain region crucial for learning and memory. In the new study, Roopra and his team found that adult mice with the same mutation show a normal degree of LTD. Instead of relying on mTOR signaling, however, these adult mutant mice normalized their LTD through increased activation of the metabotropic glutamate receptor 5 (mGluR5) and extracellular signal-regulated kinases (ERK) signaling pathways.

While activity in the mGluR5 and ERK pathways decreased in normal mice as they become adults, these pathways remained very active in adult mutant mice. The findings suggest that adult mice with the TSC2 mutation achieve a normal degree of LTD by activating compensatory pathways that are not normally involved in this cellular process. When the researchers inhibited ERK and mGluR5 signaling in hippocampal cells, LTD became dependent on mTOR signaling and seizure-like activity in the cells decreased.

Roopra and his team next tested whether the mGluR5 pathway also underlies behavioral problems that characterize TSC. The investigators placed normal and mutant mice in a pool of water with a platform hidden just below the surface. Once the mice learned the location of the platform, the researchers moved it to another spot. Compared to normal mice, mutant mice were much more likely to swim to the original location of the hidden platform. This behavior resembles repetitive, autistic-like patterns that are common among individuals with TSC. Treatment with an mGluR5 inhibitor corrected the behavioral abnormality in mutant mice, enhancing their ability to find the hidden platform in new locations. Taken together, the findings suggest that abnormalities in ERK and mGluR5 signaling are at the root of a variety of TSC symptoms.

By revealing that the signaling pathways affected by TSC change with age, the study sheds new light on the complexity of the disorder, reconciles conflicting findings from different laboratories about the role of the mTOR pathway in TSC, and highlights the importance of characterizing age-dependent effects to reach more reliable conclusions. The work also suggests that disruptions in the mGluR5 pathway may be a common mechanism underlying a range of neurodevelopmental disorders, including fragile X syndrome—the most common known cause of inherited intellectual disability. Moreover, the findings suggest that drugs targeting the mGluR5 and ERK signaling pathways could represent a novel and promising strategy to treat seizures and behavioral problems in TSC patients.

**Potter WB, Basu T, O'Riordan KJ, Kirchner A, Rutecki P, et al. (2013) Reduced Juvenile Long-Term Depression in Tuberous Sclerosis Complex Is Mitigated in Adults by Compensatory Recruitment of mGluR5 and Erk Signaling. doi:10.1371/journal.pbio.1001627**

## Abbreviations

ERK: extracellular signal-regulated kinases
TSC: tuberous sclerosis complex
mGluR5: metabotropic glutamate receptor 5
mTOR: rapamycin
LTD: long-term depression

